# A review of the genetics and epigenetics of central precocious puberty

**DOI:** 10.3389/fendo.2022.1029137

**Published:** 2022-12-02

**Authors:** Joshua Moise-Silverman, Lawrence A. Silverman

**Affiliations:** ^1^ New York Medical College, School of Medicine, Valhalla, NY, United States; ^2^ Division of Pediatric Endocrinology Goyreb Children’s Hospital – Atlantic Health System, Morristown, NJ, United States

**Keywords:** central precious puberty, genetics, epigenetic, GnRH, kisspeptin

## Abstract

Gonadotrophin dependent sexual precocity, commonly referred to as central precocious puberty (CPP), results from a premature reactivation of the hypothalamic-pituitary-gonadal (HPG) axis before the normal age of pubertal onset. CPP is historically described as girls who enter puberty before the age of eight, and boys before the age of nine. Females are more likely to be diagnosed with idiopathic CPP; males diagnosed with CPP have a greater likelihood of a defined etiology. These etiologies may include underlying CNS congenital defects, tumors, trauma, or infections as well as environmental, genetic, and epigenetic factors. Recently, genetic variants and mutations which may cause CPP have been identified at both the level of the hypothalamus and the pituitary. Single nucleotide polymorphisms (SNPs), monogenetic mutations, and modifications of the epigenome have been evaluated in relationship to the onset of puberty; these variants are thought to affect the development, structure and function of GnRH neurons which may lead to either precocious, delayed, or absent pubertal reactivation. This review will describe recent advances in the field of the genetic basis of puberty and provide a clinically relevant approach to better understand these varying etiologies of CPP.

## Introduction

Gonadotropin-dependent sexual precocity, more commonly referred to as central precocious puberty (CPP), results from the premature reactivation of the hypothalamic-pituitary-gonadal (HPG) axis (1). While data suggest that the age of onset of puberty has decreased over the past half-century, clinically, the appearance of breast development before the age of eight in girls or testicular enlargement before the age of nine in boys is generally considered precocious (2).

The mechanisms of reactivation of the HPG axis and entry into puberty are complex and incompletely understood. Initiation of both normally timed and precocious puberty involves the pulsatile release of GnRH, with subsequent increases in both pulse amplitude, and frequency as the process progresses. Increased levels of GnRH result in the secretion of luteinizing hormone (LH) and follicle stimulation hormone (FSH); this then leads to the production of gonadal sex steroids and development of secondary sexual characteristics (3).

Puberty is neither a linear process nor a single event; progression through puberty requires the interplay of various genetic and epigenetic factors and dependent on normal Hypothalamic - Pituitary anatomy.

## Physiology of puberty

**Figure 1:**
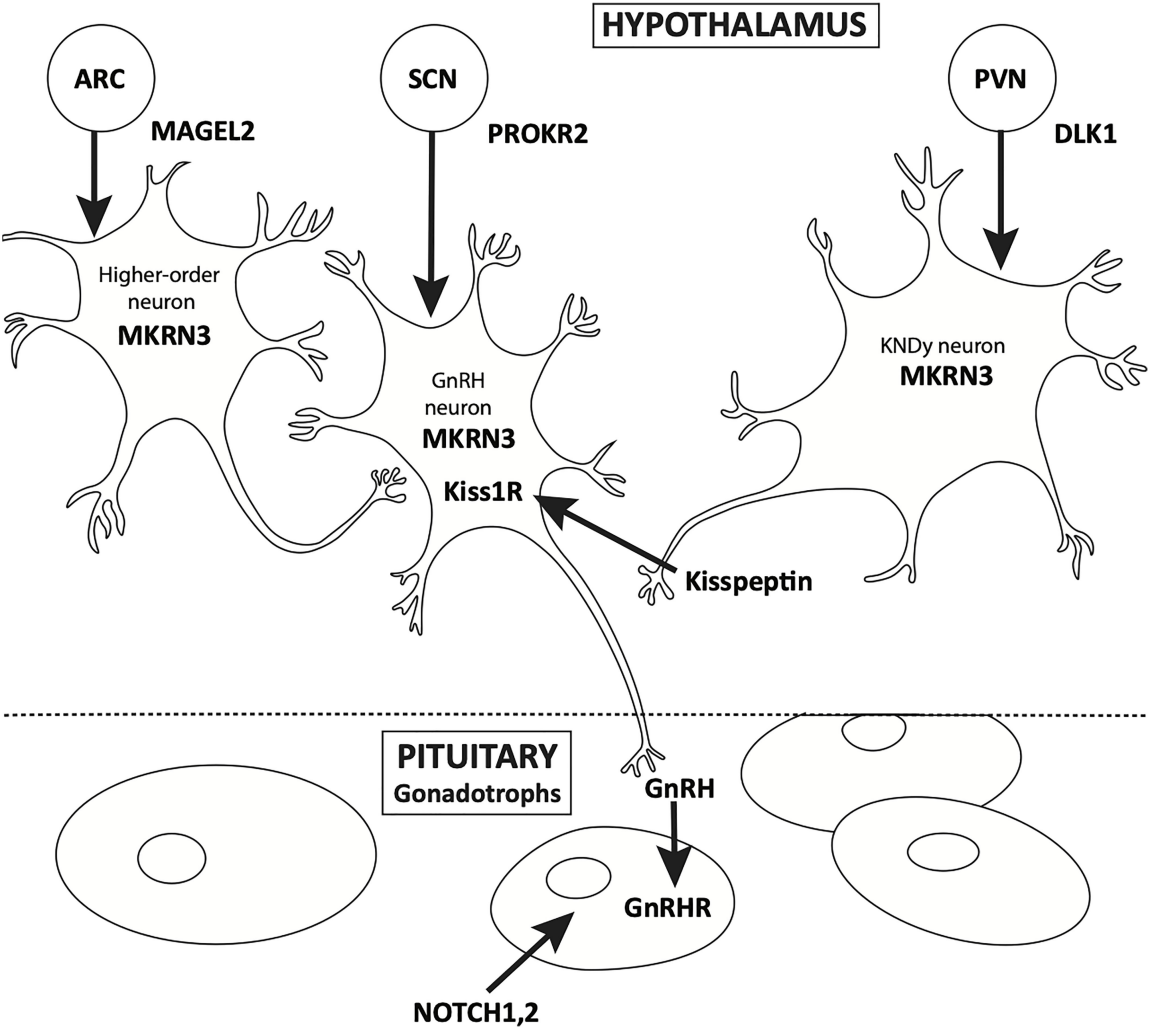
Location of expression of genes associated with CPP.

Puberty is a continuum. HPG activation occurs in early fetal life, and is integral to normal *in-utero* male sexual development. Soon after birth, the “mini-puberty of infancy” ensues, followed by the “juvenile pause” and ultimately, the reactivation of the axis, usually after the age of eight in girls and nine in boys. Over the past 50 years, the physiology of pubertal onset has been well described, starting with the studies of Knobil (4). The physiology of GnRH pulse generator, located in the arcuate nucleus of the hypothalamus, has been well characterized from fetal to adult life by elegant physiologic studies (**Figure 1**). Additionally, *in vitro* evaluation of immortalized GnRH neurons show that these cells have an endogenous rhythmicity and periodicity of GnRH production (5). While the trigger for the onset of puberty has yet to be fully described, it is well known that anatomic insults and other gross physiologic perturbations can lead to an early reactivation of the HPG axis; it is only more recently that the genetic underpinnings of this reactivation have come to be more fully understood (6, 7).

## Epidemiology

While not rare, CPP evades comprehensive epidemiological characterization. Conservative estimates suggest the incidence of CPP may be as common as 1 in 5000 while more modest definitions estimate an incidence of 1in 10,000 (8). Research utilizing data from the Danish National Patient Registry indicates the prevalence of CPP in females is nearly ten times greater than in males - 0.2% of females versus 0.05% of males (9). Despite geographic variation, most epidemiological research confirms that CPP is significantly more common in females. Furthermore, females are more likely to receive a diagnosis of idiopathic CPP, while historically an underlying anatomic or pathologic cause of CPP has been identified in up to fifty percent of males who carry this diagnosis (10); more recent surveys suggest that this percentage of organic CPP may be decreasing over time, and these boys generally present at a younger age. It is unclear if geographic, and hence environmental (11), factors are partially responsible for this possible demographic shift

CPP may be associated with a variety of co-morbid and co-occurring disorders. Individuals diagnosed with CPP are at a statistically greater risk of developing polycystic ovarian syndrome, breast cancer, and insulin resistance. Additionally, due to age-associated body image concerns, there appears to be a greater likelihood of concurrent, and possibly future, psychologic stresses (12, 13). Thus, diagnosis and treatment of CPP have both near and longer-term impacts on future health and wellbeing.

Causes of CPP include anatomic, metabolic, environmental, nutritional, geographic, and genetic factors (**Table 1**). Large, population based studies suggest that up to 50- 70% of the variation in pubertal timing is associated with genetic variation (14). The past fifteen years have seen a marked increase in the discovery and characterization of both normally functioning genes and pathologic variants associated with CPP. GWAS and other genetic technologies have further increased the discovery of genetic loci associated with pubertal timing.

**Table 1:** Genes associated with central precocious puberty.

Gene	Protein	Primary Site(s) of Expression	Mechanism(s) responsible for CPP
KISS1	Kisspeptin	Hypothalamus -arcuate *KISS1* neurons	Stimulatory - increased expression, decreased degradation, increased hydrophobic character
GPR54	Kisspeptin Receptor	Hypothalamus GnRH - neurons	Stimulatory - increased stability, increased recycling to plasma membrane, decreased degradation
MKRN3	Makorin Ring Finger Protein 3	Hypothalamus - Paraventricular nucleus, mamillary body	Stimulatory (loss of inhibition)– decreased binding affinity, decreased protein stability, loss of ubiqutin ligase activity
MAGEL II	Melanoma-Antigen-Subfamily-Like-2 Protein	Hypothalamus, pituitary	Loss of function – decreased protein stability
NOTCH 1	Notch Receptor 1	Pituitary	Loss of function - decreased expression
NOTCH 2	Notch2	Pituitary	Mechanism unknown
DLK1	Protein delta homolog1	Hypothalamus	Loss of expression – partial deletion, decreased expression
NROB1	Dosage-sensitive sex reversal, adrenal hypoplasia critical region, on chromosome X, gene 1 protein	Hypothalamus, adrenal cortex, pituitary, and gonads	Compound heterozygote – mechanism unknown
PROKR2	Prokineticin receptor 2	Hypothalamus – GnRH neurons	Gain of function – increased ligand sensitivity
PCNT	Pericentrin	Hypothalamus, various non-endocrine structures	Mechanism unknown
LIN28A/B	LIN28	Pluripotent cells of CNS	Mechanism unknown

## KISS1

Kisspeptins are a family of proteins encoded by *KISS1*(1q32). This four-exon gene is translated into the 145 amino acid propeptide kisspeptin-145, following translation kisspeptin-145 is proteolytically processed into kisspeptin 54, which is further processed into kisspeptin-14,13 and 10. All four kisspeptin proteins – 10,13,14,54, - exhibit a similar affinity for the kisspeptin receptor which is alternatively referred to as GPR54 (15). Kisspeptin is released with two co-transmitters - neurokinin B (NKB) and dynorphin (Dyn) – these proteins will be discussed in a later section.

Non-human primate studies show that kisspeptin is expressed in a variety of tissues; these additional animal and human studies indicate neural expression of *KISS1* is important for the initiation and maintenance of puberty (16). Under normal circumstances *KISS1* is expressed by neurons located in the infundibular nucleus, the site of estrogen-induced negative feedback of the HPG axis, and the medial preoptic area (mPOA), a site of estrogen positive feedback (17). Sexually dimorphic responses to estrogen are observed in the mPOA; in females, estrogen stimulation of this region results in an LH surge while in males this surge does not occur (18).

Kisspeptins are responsible for orchestrating the pulsatile release of GnRH. Thus, dysfunctional kisspeptin signaling can lead to either precocious or delayed puberty (19). While increased kisspeptin levels cannot be used to definitively define CPP, elevated serum levels may be observed in patients with CPP (18). Pathogenic variants of *KISS1* can result in 1) decreased degradation of kisspeptin 2) premature increased expression of kisspeptin due to variations in either the five prime or three prime untranslated region (UTR) 3) defects in proteolytic processing and subsequent changes to the affinity of kisspeptin for the kisspeptin receptor, all of which increase kisspeptin levels and subsequent activation of GnRH neurons.

Variants which increase the half-life of kisspeptin are rare and the exact mechanisms have yet to be elucidated. A 2010 publication described CPP in an individual with the amino acid substitution p.P74S. This variant results in the loss of a proline residue in an N-terminal PEST sequence, a region of the kisspeptin peptide needed for rapid targeting and degradation by proteolytic enzymes (20). Decreased breakdown of kisspeptin peptides was theorized to increase its half-life, resulting in CPP.

SNPs located within the UTR of *KISS1* are associated with both CPP and idiopathic hypothalamic hypogonadatropism (IHH). Polymorphisms linked to CPP are thought to increase the transcriptional efficiency of *KISS1*; the mechanisms of action have yet to be elucidated (21).

A third proposed mechanism of *KISS1*-related CPP is that of increased affinity for the kisspeptin receptor (KISS1R). Increased affinity can be due to 1) SNPs of *KISS1* or 2) SNPs of *GPR54* – the kisspeptin receptor. Interestingly, SNPs of *KISS1* associated with hypogonadotropic hypogonadism indicate that SNPs which decrease the hydrophilicity of kisspeptins may lead to delayed puberty; such SNPs have been identified and characterized by in-silico analysis, further *in-vitro* work is needed to corroborate in-silico models (22).

Mutations and variations of *KISS1* are increasingly detected *via* GWAS and targeted sequencing approaches; case reports suggest an expected response to GnRHa therapy of CPP in which variations of *KISS1* are pathogenic.

## KISS-R

*GPR54* (19p13.3) encodes the transmembrane kisspeptin receptor (KISS1R). The binding of kisspeptins to this 398 amino acid transmembrane G-protein coupled receptor results in the activation of phospholipase C *via* a Gaq-mediated signaling mechanism (17). Downstream signaling results in the pulsatile release of GnRH. All kisspeptin peptides contain the RMRFamide motif (Phe-Met-Arg-Phe-NH2) and exhibit similar affinities for KISS1R (15). Neuropeptides and artificial ligands with the same or similar motifs can agonize KISS1R; stimulation *via* exogenous ligands is weaker than stimulation *via* endogenous ligands (15). At the writing of this paper, the only known KISS1R antagonists are exogenous peptides.

The first *GPR54* variants to be identified and characterized were loss of function mutations resulting in IHH, leading to the inactivation of downstream signaling cascades; recent studies have identified several pathogenic variants which lead to CPP *via* increased activation of KISS1R and increased downstream signaling (18). A study performed in 2011 identified the autosomal dominant missense mutation Arg386Pro as a cause of idiopathic CPP (23). The change of a single amino acid residue decreases the degradation and internalization of KISS1R, which is summarily recycled to the cell surface and ultimately increases stimulation of the kisspeptin signaling pathway (23).

An evaluation of a cohort of Korean females identified several SNPs of *GPR54* which lead to the development of CPP. Of the seven identified SNPs two are associated with significantly decreased age of pubertal onset (24). The missense variant c.1091T>A results in p.Leu364His; this variant is associated with the development of CPP. At the writing of this paper molecular characterization of this SNP has not been completed, and its mechanism is unknown.

A second SNP identified by Oh and colleagues, c.738+64G>T, did not modify the amino acid sequence of KISS1R. This SNP is located within the fourth intron of *GPR54* and is hypothesized to cause CPP *via* the change in function of the regulatory mechanisms that have yet to be elucidated (24). Work completed in 2019 by Ghaemi et al. identified additional SNPs within *GPR54* that were present in the genomes of probands with familial CPP. The underlying molecular mechanism of these SNPs, c.24A > T in 13 subjects, c.1091 T > A in 16 subjects, and c.492C > G remain unstudied at this time (25). Finally, a 2020 study identified an SNP located in the 5’UTR of *GPR54* mRNA as a potential cause of CPP, further evaluation is needed to confirm this hypothesis (26).

Individuals with *GPR54 variants* or SNPs represent a small subset of CPP patients. Similar to those with *KISS1* dysfunction, they can be successfully treated with GnRH analogs.

## MKRN3

*MKRN3* is a maternally imprinted gene, only paternal alleles are expressed. This intronless gene is located within the Prader Willi Syndrome locus of chromosome 15 and encodes the E3 ubiquitin ligase makorin ring finger protein 3 (MKRN3). Variants of *MKRN3* are commonly observed in patients with familial CPP (26).

In healthy individuals, MKRN3 is predicted to prevent the premature reactivation of the HPG axis by acting as a negative regulator of hypothalamic gene expression. In hypothalamic neurons, MKRN3 binds to and represses both the kisspeptin and neurokinin B gene promoters, preventing the expression of genes associated with HPG reactivation. Additional murine research indicates MRKN3 epigenetically silences the expression of *GNHR1* by preventing the interaction of MDBH3 with the promoter of *GNHR1* (27). Before the onset of puberty MRKN3 levels greatly decrease, further highlighting its potential role as an inhibitor of puberty-related gene expression (28).

MKRN3 has been shown to interact with 81 proteins, 21 of which are implicated in the timing of HPG axis reactivation (29). Due to the inheritance patterns of *MKRN3*, paternally inherited pathogenic variants often lead to familial CPP (fCPP); *MKRN3* dysfunction is thought to contribute to up to 30% of fCPP cases (30, 31). *MKRN3* loss of function variants are the most common cause of CPP in individuals with fCPP. These changes are thought to decrease inhibition of GnRH neurons *via* a variety of mechanisms: 1) decreased binding affinity due to zinc finger dysfunction, 2) decreased stabilization of peptides *via* MKRN3-target protein interaction 3) increased expression of genes related to puberty, and 4) decreased ubiquitin ligase activity (32). Currently, over forty clinically relevant missense, nonsense and frameshift variants of *MKRN3* have been identified. In addition to loss-of-function, recent research has identified pathogenic variants associated with regulatory regions of *MKRN3.* The molecular mechanisms associated with dysregulation of *MKRN3* expression have yet to be elucidated (33). One possible explanation is due to mutations of the 3’-UTR. These variants prevent miR-30 binding, thus altering transcript stability.

In addition to fCPP, spontaneous variants of *MKRN3* have been identified in several individuals. Although spontaneous variants are rare they should be considered in the setting of female patients diagnosed with apparent idiopathic CPP. Patients diagnosed with both idiopathic and familial *MKRN3*-associated CPP respond well to treatment with GnRH analogs.

Due to *MRKN3’s* location on the PWS region of chromosome 15, loss of *MKRN3* or damage to this gene should be considered in patients with Prader Willi Syndrome patients who develop symptoms of CPP. While most patients with PWS display delayed or absent puberty, several reports indicate that CPP can rarely occur (34–36).

Differential methylation and imprinting of *MKRN3* is primarily controlled by a two-part imprinting control center located upstream of *MKRN3.* This region contains the SNPRN promoter, the PWS imprinting control center (IC) and the AS IC (37). Research has shown the PWS IC is bidirectional and controls expression of the maternal *UBE3A via* expression of antisense RNA expression (38). Thus, loss of PWS-IC expression increases methylation of the paternal genes and leads to PWS. Loss of the maternal allele due to deletion or inactivation leads to the development of AS. Further discussion of the molecular mechanism which underlie differential methylation of the PWS region of chromosome 15 is beyond the scope of this paper. For the purposes of CPP it is important to recognize PWS-IC regulates expression of paternally expressed genes *via* long range chromatin interaction, the site of chromatin interaction lies between *MKRN3* and *MAGEL2*. Thus mutations which flank these genes can affect chromatin interactions and expression of genes associated with PWS, AC and CPP (37, 38). A deeper understanding of the molecular dynamics and three-dimensional interaction of the PWS-IC and chromatin interaction sites located on chromosome 15 may illustrate additional genetic and epigenetic phenomena related to CPP.

## MAGEL II

*MAGEL II* is a single exon gene encoding the melanoma-antigen-subfamily-like-2 protein (MAGEL II) also located within the Prader-Willi- Syndrome region of chromosome 15. *MAGEL II* is expressed in the hypothalamus and other brain tissues (39). This gene is maternally imprinted, indicating paternally inherited variants are pathogenic. Changes in *MAGEL II* are implicated in a number of rare genetic syndromes, many of which present with hypopituitarism or hypogonadism, CPP has also been reported (34, 35, 40).

While the mechanisms of *MAGEL II-*related CPP have yet to be elucidated, current research hypothesizes that the loss of several genes within the PWS region of chromosome 15 can lead to CPP, as opposed to the more common phenomena of delayed puberty. A potential explanation for this phenomenon is due to the MRKN3-MAGEL II interaction. The E3 ubiquitin ligase encoded by *MKRN3* is enhanced and stabilized by MAGEL- II; thus, mutations that decrease the stability of MKRN3 may result in early reactivation of the HPG axis.

## HERC2

*HERC2* encodes the E3 ubiquitin ligase HERC2. This gene is located on the 15^th^ chromosome and is associated with PWS, Angelman Syndrome, and Oculocutaneous Albinism, three syndromes which have been noted to be comorbid with pubertal disorders (41). In addition to ubiquitination, *HERC2* contains an intronic regulatory element known to inhibit the promoter of *OCA2.* Deletion of *OCA2* is also associated with PWS, Angelman syndrome, and Oculocutaneous Albinism type 2.

## DLK1

*DLK1* is a maternally imprinted and paternally expressed gene located on the 14^th^ chromosome. *DLK1* encodes protein delta homolog1 (PDH1). Sites of gene expression include the hypothalamus, pituitary gland, and adrenal glands. Murine and rodent enrichment studies demonstrate high levels of PDH1 in orexin- and dynorphin-containing neurons; dynorphin is coexpressed with kisspeptin (42).

PDH1 is associated with the delta notch signaling pathway, suggesting that this gene and its protein product are important for the normal development and homeostasis of various structures. During development, *DLK1* ensures normal development of kisspeptin neurons and timely maturation of the HPG axis. Recent case studies have illustrated the relationship between CPP and *DLK1*. A study involving a family of five females diagnosed with CPP identified a complex partial deletion of chromosome 14, the 5’UTR and first intron were deleted, and downstream structures remained (43). These patients had undetectable levels of circulating PDH1.

Additional studies have attempted to identify genetic causes of idiopathic CPP due to variations of *DLK1;* three novel loss of function frameshift mutations were identified in the 2019 study performed by Gomes et al. (44) Characterization of these five probands indicates pathogenic variations of *DLK1* are rare but should be considered in the setting of female patients with a history of familial CPP.

Temple syndrome (TS), an imprinting disorder caused by the loss of 14q32.2 due to maternal uniparental disomy, epimutations or micro deletions is a known cause of GnRHa responsive CPP. In addition to delay of puberty, these patients display delayed gross motor development, hypotonia, intellectual disabilities, and other symptoms (45). PWS like symptomology is also common. Given the chromosomal region associated with TS and the development of CPP, genes located at 14q32.2, mutations, mis-methylation or imprinting defect of *DLK1* and *MEG3* should be considered in the setting of idiopathic CPP.

Due to maternal methylation, paternally inherited deletions of *DLK1* are a rare but known cause of CPP (43). The mechanism underlying decreased age of pubertal onset is not fully defined at this time. Several papers hypothesis that *DLK1* related CPP is due to upregulation of kisspeptin neuron differentiation (46, 47). In brief absence of DLK1 in developing hypothalamic neurons results in increased NOTCH signaling. Increases in NOTCH1 and 2 result in increased expression of target genes, these genes *HES1*, *HEY1* and others transcriptional repressors results in differential expression of genes related to kisspeptin neuron differentiation. Thus abnormal methylation or imprinting of DLK1 increases kisspeptin neuron neurogenesis, which may underlie DLK1 related CPP (46–48).

At this time, mechanisms underlying differential methylation and expression of maternal and paternal *Dlk1-Dio3* gene cluster are known to be controlled by *IG-DMR*, this imprinting control region exhibits unique paternal and maternal patterns of methylation (49). Maternal *IG-DMR* is relatively unmethylated, thus acting as a CIS enhancer of the noncoding *Rtlas, Rian, Mirg* and *Gtl2* expression (49). Knocking out the maternal *IG-DMR* is not compatible with life. Conversely, paternally imprinted *IG-DMR* is methylated. Thus, paternal copies of the *Dlk1-Dio3* cluster do not express the aforementioned ncRNAs, instead expressing *Dlk1, Rtl1* and *Doi* (49). The differential methylation and expression patterns of the *Dlk-Dio3* cluster and the *IG-DMR* highlight genetic and epigenetic phenomena that require additional study in order to determine if a relationship to CPP exists.

## NOTCH 1 and 2

Given the role of DLK1 in the notching signalling pathway, researchers have attempted to identify cases of CPP related to pathogenic variants of the Notch genes. These genes are a highly conserved family of developmentally active receptors involved in the normal development of the nervous, cardiac and endocrine systems (50). Once a ligand binds to a Notch receptor, the receptor is cleaved by ADAMs (a disintegrin and metalloproteinase), and downstream signaling follows. Notch signaling cascades are mediated by E3 ubiquitin ligases. While the exact mechanisms linking notch signaling and CPP remains to be understood, the genomes of several probands diagnosed with CPP have been found to contain pathogenic variations within *NOTCH1* and *NOTCH2*(50–52)

*NOTCH1*, located on chromosome 9, encodes the single-pass transmembrane receptor Notch1. A case report published in 2016 identified a proband diagnosed with 22q13 gene duplication syndrome, duplication of *NOTCH1*, and CPP (52). Multiple authors have hypothesized duplication of *NOTCH1* resulted in increased gene dosage, thus modifying the developmental trajectory of kisspeptin neurons and leading to the development of CPP (50). The CPP was successfully treated with a GnRH analog.

Like *NOTCH1*, *NOTCH2*, located on chromosome one, encodes the single-pass transmembrane receptor Notch2. Notch2 has several ligands, including PDH1. This gene is expressed in a variety of developing tissues; in adults, high levels of expression are detected in the hypothalamus, pituitary, and other organs.

The exact mechanism of Notch2’s effect on the reactivation of the HPG axis is unknown. A 2020 case report by Lee et al. indicates that Notch2 influences the timing of puberty *via* interaction with the protein product of *HERC2* (51) In this study, two siblings with apparently idiopathic CPP were found to harbor two heterozygous missense variations: p.Leu15Phe in NOTCH2 and p. Arg4081His in HERC2; the parents of these children each carried a single variant but did not have a history of precocious puberty (51). The compound effect of these two SNPs indicates a normal function of *NOTCH2* and *HER2* may be needed to allow for normal timing of puberty.

## NROB1

In the setting of CPP variations of *NROB1* are a rare cause of CPP. *NROB1* encodes the dosage-sensitive sex reversal, adrenal hypoplasia critical region, on chromosome X, gene 1 protein (DAX1). DAX1 is expressed in the hypothalamus, adrenal cortex, pituitary gland, and gonads. This nuclear receptor is primarily responsible for the negative regulation of other nuclear receptors. Unlike many nuclear receptors, DAX1 lacks a DNA binding motif, thus it exerts its effects by binding to and decreasing the activity of various proteins, including SRY, SF1, as well as additional genes involved in the regulation and development of a variety endocrine cells and tissues (53).

Variants of *NROB1* lead to a variety of endocrine disorders, primarily hypogonadotropic hypogonadism and X-linked adrenal hypoplasia. Recent investigations indicate changes of *NROB1* can result in X linked AHC with CPP. Multiple reports have identified novel mutations of *NROB1* that lead to both X-linked adrenal hypoplasia and CPP (54, 55). Interestingly, several studies report that certain variants of *NROB1* resulting in CPP can be managed *via* the treatment of AHC, as opposed to treatment with a GnRH agonist. Zhang and colleagues successfully utilized hydrocortisone and fludrocortisone to treat an 11-month-old patient with heterozygous *NROB1* mutations, AHC and CPP. Following administration of the aforementioned medications, secondary sexual characteristics reverted, and LH and testosterone levels also decreased to age-appropriate levels. Two-year follow-up confirmed successful management of CPP (56).

*NROB1* variants remain a rare but important cause of CPP in patients diagnosed with AHC. At this time the mechanisms underlying *NROB1*-related CPP are unknown. Yank and colleagues hypothesize weak interactions between DAX-1 and SF1 lead to prolonged activation of hypothalamic NO synthetase and the development of CPP. It is unclear if the findings of Yang et al. can be generalized to various pathogenic variants of the *NRBOB1* gene (57).

## PROKR2

*PROKR2*, located on chromosome 20, encodes the dimeric 384 ammino acid GPCR prokineticin receptor 2. This receptor is expressed on the surface of developing hypothalamic GnRH neurons; activation *via* several ligands results in the promotion of GnRH secretion. Variations of *PROKR2* are known to result in hypogonadotropic hypogonadism, gynecomastia, and idiopathic anosmia, recent reports have highlighted a gain of function mutation resulting in CPP (58).

The majority of patients with *PROKR2*-related CPP are female. Work performed by Fukami and colleagues identified a heterozygous frameshift variant which resulted in the generation of an mRNA that was not subject to nonsense-mediated decay. *In-vivo* studies indicate this led to increased ligand sensitivity and increased downstream signaling when variant and wild-type proteins were coexpressed (59). Interestingly this variant was also identified in the probands mother, but symptoms of CPP were not identified. Following the work of Fukami, two studies attempted to identify the prevalence of *PROKR2* variations in cohorts of 25 and 31 unrelated females with idiopathic CPP; neither study identified additional pathogenic variants of interest, thus suggesting that *PROKR2* mutations are a rare cause of CPP (60, 61).

## LIN28B/A

*LIN28B* is a protein-encoding gene located on chromosome six. Several GWAS studies have identified SNPs of *LIN28B* in female and male patients with idiopathic CPP (13, 62–64). These studies used age of menarche, pubic hair development, age at voice breaking, and other measures as surrogate measures for pubertal onset. Research efforts focusing on individual patients or cohorts of patients with CPP have identified several rare *LIN28B* variants; these variants are not commonly involved in the development of CPP (65).

*LIN28B* encodes LIN28; this protein is involved in the degradation and negative regulation of the miRNA let-7 (66). The mechanism by which *LIN28B* SNPs affect the timing of puberty remains unclear. More research is needed to determine the clinical utility of these SNPs and their relationship to pubertal onset.

## CPP and the methylome

Studies of DNA methylation in pre or post pubertal individuals with or without CPP has allowed researchers to identify the epigenetic mechanisms which underlie the activation and matience of the HPG axis. In female patients there are over 120 chromosomal sites that display different methylation patterns before and after puberty. In postpubertal females these sites are primarily hypermethylated and located on the X chromosome (67). In females with CPP, there are an increased number of hypermethylated CpG islands, this hypermethylation is present in both pre and post pubertal individuals.

*ZFP57* encodes zinc fingered protein 57, a DNA binding protein involved in the methylation of *MKRN3.* Hypomethylation of the *ZFP57* promoter is associated with both the initiation and maintenance of puberty; increased levels of hypomethylation are observed in the hypothalamus of non-human primates and the blood cells of female humans (68). *ZFP57* knockout models display higher levels of *MKRN3* expression, a negative repressor of hypothalamic gene expression (68).

## Conclusion

The concept that puberty begins in the brain, secondary to both an increase in “stimulatory” factors and a decrease in “inhibitory” factors, which rely on the interplay of myriad genes and their protein products, has been elucidated and evolved over the past 50 years. Central precocious puberty may have multiple etiologies: developmental, anatomic, environmental, genetic, and epigenetic. With the continued burgeoning of population based genetic studies, as well as the specific evaluation of kindreds with fCPP, a greater understanding of the individual genes responsible for the onset of puberty is coming in to further focus. A resultant understanding of what truly controls the onset of puberty may be close at hand. It is likely that with the expanded use of genetic tools in the clinic, this understanding will further expand, to the point that what controls not only the timing, but also the tempo of pubertal progression will be understood. Perhaps in the not-too-distant future, true genotype/phenotype correlations will help drive therapeutic considerations.

## Author contributions

JM-S performed the literature review and authored this manuscript, LS content and editorial guidance. All authors contributed to the article and approved the submitted version.

## Acknowledgments

Mona Chen provided assistance with the development of **Figure 1**.

## Conflict of interest

 Author LS is a consultant to ENDO Pharma, Tolmar Pharmaceuticals, Enteris BioPharma, Myovant Sciences.

The remaining author declares that the research was conducted in the absence of any commercial or financial relationships that could be construed as a potential conflict of interest.

## Publisher’s note

All claims expressed in this article are solely those of the authors and do not necessarily represent those of their affiliated organizations, or those of the publisher, the editors and the reviewers. Any product that may be evaluated in this article, or claim that may be made by its manufacturer, is not guaranteed or endorsed by the publisher.
